# Microbiome analysis reveals potential for modulation of gut microbiota through polysaccharide-based prebiotic feeding in *Oreochromis niloticus* (Linnaeus, 1758)

**DOI:** 10.3389/fphys.2023.1168284

**Published:** 2023-06-08

**Authors:** Asit Kumar Bera, Hemanta Chowdhury, Sandeep Ghatak, Ramesh Chandra Malick, Nabanita Chakraborty, Hirak Jyoti Chakraborty, Himanshu Sekhar Swain, M. A. Hassan, Basanta Kumar Das

**Affiliations:** ^1^ Central Inland Fisheries Research Institute (ICAR), Bārākpur, India; ^2^ The ICAR Research Complex for North Eastern Hill Region (ICAR RC NEH), Umiam, India

**Keywords:** Fish, *Oreochromis niloticus*, gut, microbiome, prebiotics, plant polysaccharides

## Abstract

Characterization and functional profiling of the gut microbiota are essential for guiding nutritional interventions in fish and achieving favorable host-microbe interactions. Thus, we conducted a 30 days study to explore and document the gut microbial community of *O. niloticus*, as well as to evaluate the effects of a polysaccharide-based prebiotics with 0.5% and 0.75% Aloe vera extract on the gut microbiome through genomic analysis. The V3–V4 region of 16S rRNA was amplified and sequenced using Illumina HiSeq 2500, resulting in 1,000,199 reads for operational taxonomic unit (OTU) identification. Out of 8,894 OTUs, 1,181 were selected for further analysis. Our results revealed that Planctomycetes, Firmicutes, Proteobacteria, Verrucomicrobia, Actinobacteria, and Fusobacteria were the dominant phyla in both control and treatment samples. Higher doses of prebiotics were found to improve Planctomycetes and Firmicutes while decreasing Proteobacteria and Verrucomicrobia. We observed increasing trends in the abundance of Bacilli, Bacillaceae, and *Bacillus* bacteria at the class, family, and genus levels, respectively, in a dose-dependent manner. These findings were consistent with the conventional colony count data, which showed a higher prevalence of *Bacillus* in prebiotic-supplemented groups. Moreover, predicted functional analysis using PICRUSt indicated a dose-dependent upregulation in glycolysis V, superpathway of glycol metabolism and degradation, glucose and xylose degradation, glycolysis II, and sulfoglycolysis pathways. Most of the energy, protein, and amino acid synthesis pathways were upregulated only at lower doses of prebiotic treatment. Our findings suggest that the gut microbiome of *O. niloticus* can be optimized through nutritional interventions with plant-based polysaccharides for improved growth performance in commercial fish.

## 1 Introduction

The gut microbiota comprises a diverse group of microbes that inhabit the host’s intestinal environment, thereby influencing host physiology (Feng et al., 2018). In terrestrial and aquatic vertebrates, the microbial ecosystem of the gastrointestinal tract involves host-microbe and microbe-microbe relationships that support digestion, nutrition, and health (Viney and Riley, 2014; Wang et al., 2017). The gut microbiota is crucial for gut epithelium development, enzymatic functioning, nutrient supply, and immune system stimulation (Nayak, 2010), and it is known as an extra organ due to its critical role in host growth and health (Feng et al., 2018). Dysbiosis in the gastrointestinal (GI) micro-ecosystem can lead to digestive enzyme dysfunction, gut epithelium damage, and penetration of pathogens and toxins from the lumen (Chassaing et al., 2015; Zhou et al., 2015). Researchers have become interested in the relationship between changes in the micro-ecosystem and various health problems (Qin et al., 2015). In recent years, researchers have explored the significant role of gut microbial communities in maintaining host homeostasis, growth, and disease resistance in fish. Nutritional technologies such as feed additives, probiotics, and prebiotics have been used to modulate gut flora (Hoseinifar et al., 2019; Peng et al., 2020). Probiotic supplemented diet was successfully used to achieve optimum growth performance through modulation of gut microbial community and improved digestive enzyme activity in *Labeo rohita* (Ghori et al., 2022). Use of better quality prebiotics alone or in combination with suitable probiotics have marked impact on microbiota in gut ecosystem that influence gut function and health of Atlantic salmon (Dhanasiri et al., 2023). Understanding the composition and structure of the gut microbiota and their effects on host growth and health is crucial for maintaining metabolic stability. Modulating the gut microbiota can improve host-microbe interaction and serve as a therapeutic strategy for metabolic disorders (Ojeda et al., 2016).

In this context, prebiotic substances are noteworthy as they do not directly contribute to any new flora, rather, they create a congenial environment for the favorable flora to flourish. Tilapias (*Oreochromis niloticus*) are excellent and cheap sources of animal protein worldwide. Their mode of reproduction, growth, and stress tolerance made them an ideal fish species to be intensively cultured both in enclosed and open waters (Tarnecki et al., 2017; Nicholson et al., 2019). Therefore, considering the economic importance of fish in the aquaculture industry, the functional profiling of the gut microbiota of tilapia is of utmost significance. This will help to modulate the supplements added to the feed with reduced mortality and higher yield which can promote the growth of beneficial bacteria and combat the harmful bacteria colonizing the fish gut (Ghanbari et al., 2015).

However, the exact mechanism of gut microbiota modulation by prebiotics is poorly understood in *O. niloticus* even though a few studies are available (Hasan and Banerjee, 2020; Souza et al., 2020). Of late, researchers have employed comparative microbiome analysis to evaluate the nutrition, growth, immunity, and health status of mammalian, avian, and piscine species. In our previous experiments, we observed positive growth effects of a plant-based polysaccharide prebiotic formulation (unpublished data). Therefore, to investigate the effects of the prebiotic on the gut microbiome and its prediction functionality for *O. niloticus,* we undertook the present study at two different dosage levels using a microbial community genomic approach.

## 2 Materials and methods

### 2.1 Fish rearing and feeding management

Tilapia (*Oreochromis niloticus*) fish, 200 nos, weighing 15–20 g were procured from institutional fish breeding facility, kept in fiberglass reinforced plastic (FRP) tanks, and acclimatized for 15 days. The feed was formulated and prepared in the institute’s feed laboratory, and it was provided to the experimental fish reared in FRP tanks for 30 days. The Institute Animal Ethics Committee (protocol code IAEC/2021/07; DATE: 10 12 2021), ICAR-CIFRI duly approved the entire experiment.

### 2.2 Extraction of Aloe vera

The Aloe vera polysaccharide mostly made up of acetylated glucomannan, mannose, cellulose, pectin and xylose were extracted from freshly harvested leaves using hot water extraction, followed by ethanol precipitation, according to the method described by Sun et al. (2011). After homogenization, the leaf gel, free of yellow sap (aloin), was extracted in hot water and precipitated with ethanol. The resulting white precipitate was washed with ethanol, acetone, and ether to remove impurities and then dried in a hot air oven at 70°C for 8 h, yielding an off-white powder.

### 2.3 Feeding experiment

The fish were divided into three groups, with 45 fish in each group. Each group was kept in three replicates. Group Tilapia Pre-Biotic 1 (TPB1) and Tilapia Pre-Biotic 2 (TPB2) were provided with 0.5% and 0.75% Aloe vera extract, respectively, in the feed. On the other hand, fish in Group Tilapia Control (TCON) were kept under the control diet (Table 1). Some upper and lower doses were also tried in preliminary study. But these two doses were found to be effective without altering feed composition. Feed was prepared by adding the prebiotic on top. Since the dose was below 1%, replacement of any component was not considered. After 30 days of feeding trials, three representative fish were randomly selected from each replicate and sacrificed for gut collection and further analysis.

**TABLE 1:** Diet composition (g/100g) for experimental fishes of different groups.

Ingredients	TCON	TPB1	TPB2
Soyabean meal	25.0	25.0	25.0
Rice bran	25.0	25.0	25.0
Mustard oil cake	20.0	20.0	20.0
Wheat flour	10.0	10.0	10.0
Fish meal	05.0	05.0	05.0
Groundnut oil cake	10.0	10.0	10.0
Vitamin C	01.0	01.0	01.0
BHT	0.2	0.2	0.2
Vegetable oil	1.0	1.0	1.0
Fish oil	2.0	2.0	2.0
Choline chloride	0.01	0.01	0.01
Phytase	0.01	0.01	0.01
Aloe vera extract	—	0.5	0.75

### 2.4 Sample collection and processing

The Fish were euthanized using clove oil at 50 ppm in water (Griffiths, 2000). After dissection, the whole intestine was aseptically removed and placed into sterile phosphate buffer saline (pH 7.2). The intestine samples were chopped and homogenized using a hand-held homogenizer. From each replicate, three fish were taken, and all nine samples were pooled for each group. The processed samples were used for bacterial colony counting and gut microbiome study.

### 2.5 Colony counting

The total bacterial count, *Bacillus*, and Bifidobacterium counts were determined using Nutrient Agar, *Bacillus* Medium, and Bifidobacterium Agar plates (Himedia, India), respectively. The method followed the standard protocol of tube dilution and plate counting described earlier (Wayne, 2011). Bacterial loads were expressed in cfu/gm of the whole gut sample and compared among the groups.

### 2.6 DNA isolation, 16S rRNA V3-V4-based Illumina library preparation, and sequencing

The DNA was isolated from gut samples using the DNeasyPowerSoil kit (Qiagen, United States) as per the manufacturer’s guidelines. The DNA concentration was estimated using the QubitFluorimeter (V.3.0). The V3–V4 region of the 16S rRNA was amplified using the specific V3 Forward primer, CCTACGGGNBGCASCAG, and V4 Reverse primer, GACTACNVGGGTATCTAATCC. The amplified product was checked on a 2% agarose gel, and gel purification was performed to remove non-specific amplifications. Library preparation was done using 5 ng of the amplified product from the NEB Next Ultra DNA library preparation kit. Quantification and quality estimation of the prepared library were done using the Agilent 2200 TapeStation and sequenced on Illumina HiSeq 2500 using 2*250 cycles of chemistry.

### 2.7 Quality check

The raw reads obtained from Illumina sequencing were demultiplexed and quality checked using the Fast QC program version 0.11.9 (https://www.bioinformatics.babraham.ac.uk/projects/fastqc/) under default parameters. Before bioinformatics analysis, the base quality (Phred score; Q), base composition, GC content, ambiguous bases, and adapter dimers were checked. Chimeras were then removed using the *de novo* chimera removal method, UCHIME (version 11), implemented in the VSEARCH tool.

### 2.8 Operational taxonomic units (OTU) and classification

The OTU picking and taxonomic classification were performed using the consensus V3–V4 sequences. Based on the sequence similarity, the sample pre-processed reads were pooled and clustered into OTUs using the UCLUST program (similarity cutoff = 0.97), which is available in the QIIME software (Caporaso et al., 2010). A total of 8,894 OTUs were identified from 1,000,199 reads. Out of 8,894 total OTUs, 7,713 OTUs with fewer than five reads were removed, and 1,181 OTUs were selected for further analysis. To analyze the microbial diversity within samples, Shannon and Chao1 matrices were calculated.

### 2.9 Bioinformatics analysis

To speculate on the role of microbes in host metabolism, functional profiling predictions were performed using Phylogenetic Investigation of Communities by Reconstruction of Unobserved States (PICRUSt) (Langille et al., 2013). The type of functional predictions was set to Kyoto Encyclopedia of Genes and Genomes (KEGG) orthologs. The obtained findings from PICRUSt were evaluated using versatile matrix visualization and analysis software (MORPHEUS) (https://software.broadinstitute.org/morpheus) (Starruß et al., 2014) for significant differentiation in microbial function among the fish groups due to the supplementation of prebiotics in feed. The software helps to execute the hierarchical clustering, sort and filter the discrete data based on numerous descriptive quantitative measures.

## 3 Results

**FIGURE 1:**
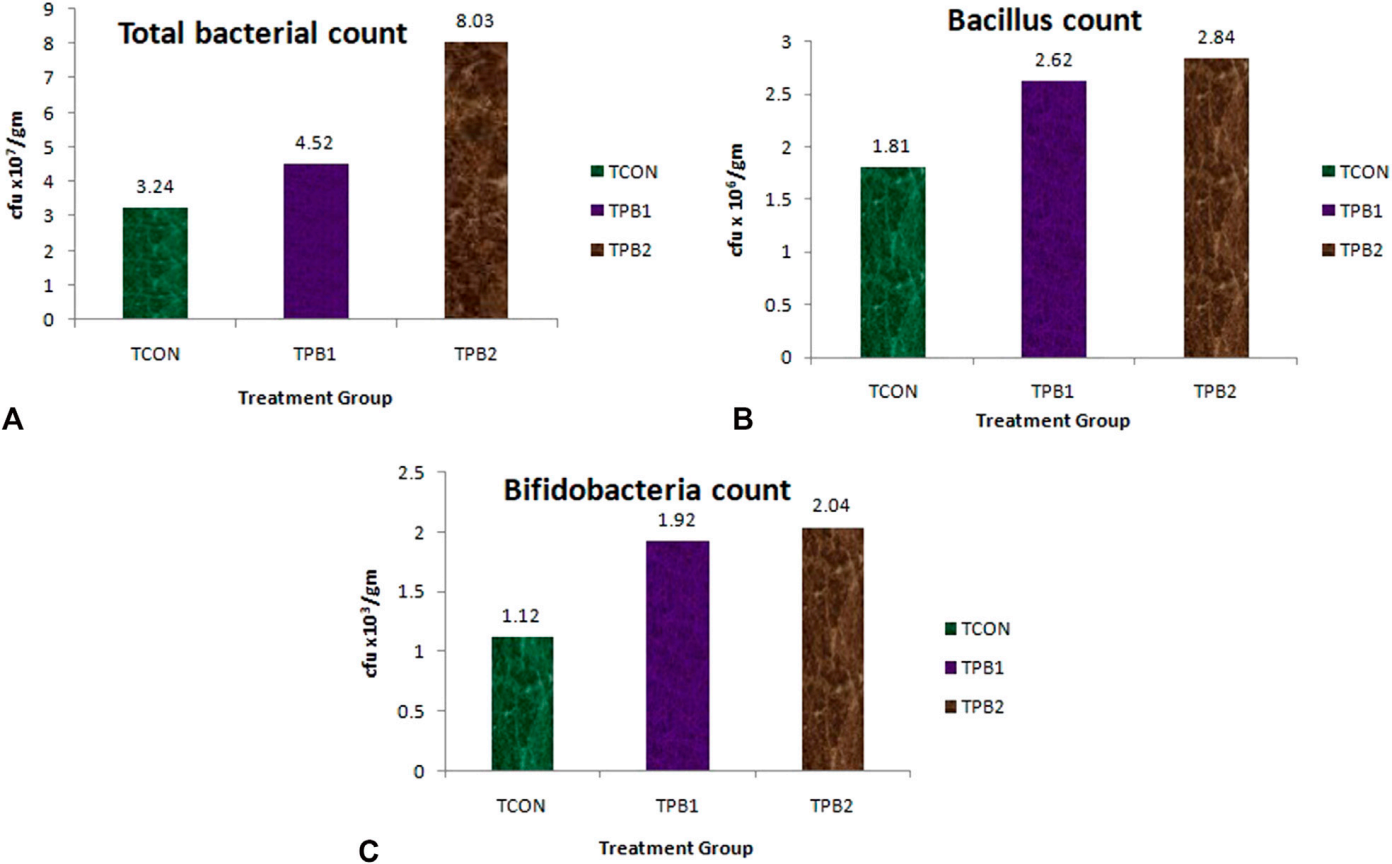
**(A)** Total bacterial load (cfu/mL), **(B)** *Bacillus* count and **(C)** Bifidobacteria count in whole gut of *O. niloticus* in different treatment group of fishes.

The total bacterial count in the control fish (TCON) was 3.24 × 10^7^ cfu/gm in the whole gut sample (Figure 1A). Treatment with plant-origin polysaccharides influenced the total bacterial count, increasing it upto 4.52 × 10^7^ cfu/gm in the treated group TPB1, and up to 8.03 × 10^7^ cfu/gm in the treated group TPB2. The *Bacillus* count was also higher in the treatment groups (Figure 1B) [1.92 × 10^6^ CFU/gm and 2.04 × 10^6^ CFU/gm in 0.5% (TPB1) and 0.75% (TPB2) prebiotic-supplemented groups, respectively] than in the control (TCON) group (1.12 × 10^6^ CFU/gm). In the control group of *O. niloticus*, the Bifidobacterium count was 1.81 × 10^3^ cfu/gm of the whole gut (Figure 1C). Moreover, both treatment doses showed it’s higher count (2.62 × 10^3^ and 2.84 × 10^3^ cfu/gm, respectively) (Supplementary Figure S1).

### 3.1 Operational taxonomic units (OTU) and classification

The process of OTU picking and taxonomy classification was conducted using pre-processed consensus V3–V4 sequences. The pre-processed reads from all samples were pooled and clustered into OTUs based on their sequence similarity using the UCLUST program (similarity cutoff = 0.97), which is available in the QIIME software. A total of 8,894 OTUs were identified from 1,000,199 reads. Out of these 8,894 OTUs, 7,713 OTUs with less than five reads were excluded, leaving 1,181 OTUs for further analysis.

#### 3.1.1 Phylum

**FIGURE 2:**
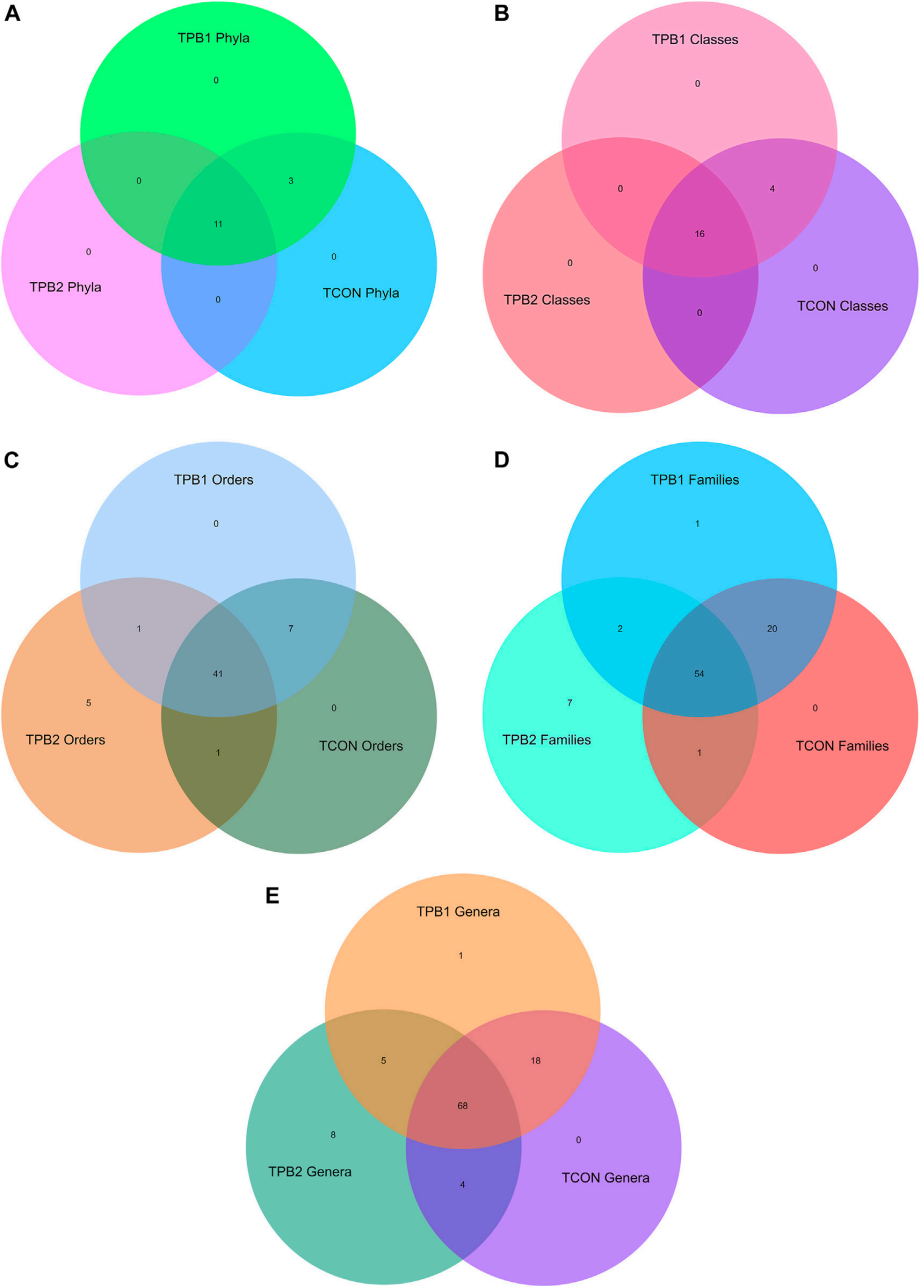
**(A)** Venn diagram of shared OTUs of bacterial phylum in gut amongst different treatment and control group fishes. **(B)** Venn diagram of shared OTUs of bacterial classes in gut amongst different treatment and control group fishes. **(C)** Venn diagram of shared OTUs of bacterial classes in gut amongst different treatment and control group fishes. **(D)** Venn diagram of shared OTUs of bacterial families in gut amongst different treatment and control group fishes. **(E)** Venn diagram of shared OTUs of bacterial genus in gut amongst different treatment and control group fishes.

Based on the top 50 hit analyses, 13 phyla were recorded in the control group of tilapia fish. The highly dominant phylum was Proteobacteria (64.78%), followed by Planctomycetes (9.77%), Actinobacteria (6.59%), Fusobacteria (5.30%), Firmicutes (3.6%), Verrucomicrobia (3.06%), Chlamydiae (1.64%), and Bacteroidetes (1.17%). Other Phyla noted were Chloroflexi, Patescibacteria, Dependentiae, Epsilonbacteraeota, and Tenericutes. The fish supplemented with a lower dose of polysaccharides (0.50%) showed an equal number of presences of the same phylum as the control group, whereas in the 0.75% supplemented group, three phyla, namely, Chlamydiae, Epsilonbacteraeota, and Tenericutes could not be detected. The abundance of Planctomycetes and Firmicutes had increased, and that of Proteobacteria had reduced in the higher dose-supplemented group of fishes (TPB2 group) (Figure 2A).

#### 3.1.2 Class

At class-level analysis, a total of 19 classes were noted in the control fishes and the lower dose-supplemented group (0.5%). Dominating classes were Alphaproteobacteria (49.35%), Gammaproteobacteria (12.72%), Planctomycetacia (9.77%), Actinobacteria (5.65%), Fusobacteria (5.30%), Verrucomicrobiae (3.06%), Deltaproteobacteria (2.70%), Bacilli (2.35%), Chlamydiae (1.64%), and Bacteroidia (1.17%). When compared to other prebiotic doses and the control group, Chlamydiae, Anaerolineae, Campylobacteria, and Mollicutes were absent in the higher dose group (TPB2). When fishes were supplemented with a higher dose of polysaccharide (0.75%), Planctomycetacia and Bacilli increased, but Gammaproteobacteria were reduced (Figure 2B).

#### 3.1.3 Order

Analyzing the order level in the top 50 hits, a total of 47 orders were recorded in control *O. niloticus* fish. The dominating orders were Rhizobiales (36.74%), Rhodobacterales (7.06%), Fusobacteriales (5.30%), Legionellales (4.0%), Pirellulales (4.0%), Micrococcales (3.53%), Verrucomicrobiales (2.82%), Gemmatales (2.23%), Reyranellales (2.23%), Pseudomonadales (2.00%), Isosphaerales (1.88%), Chlamydiales (1.64%), Planctomycetales (1.64%), Bdellovibronales (1.53%), Oligiflexales (1.17%), and Enterobacteriales (1.17%). Supplementation with prebiotics in a lower dose (0.50%) did not alter the microbial community, whereas, in a higher dose (0.75%), seven orders could not be detected. Besides, the abundance of Rhizobiales and Fusobacteriales were slightly reduced, whereas Rhodobacteriales were increased in both the supplemented groups. Supplementation with a higher dose of polysaccharide to the fish caused significant enhancement of Bacillales, Gemmatales, and Isosphaerales counts and a reduction in Reyranellales, Verrucomicrobiales, Betaproteobacteriales, and Corynebacteriales counts in comparison to the lower dosage (0.50%) and control group. Chlamydiales could not be detected in the higher-dose treated group (TPB2) fish, but the same was recorded in lower-dose-supplemented (TPB1) and control fish (TCON) (Figure 2C).

#### 3.1.4 Family

A total of 43 families were recorded in healthy (control) tilapia fishes. The most dominating family was Rhizobiales incertae sedis (17.78%), followed by Rhodobacteriaceae (7.06%), Beijerinckiaceae (5.53%), Fusobacteriaceae (5.06%), Legionellaceae (4.00%), and Pirellulaceae (4.00%). The comparison revealed that an equal number of families were seen in the TPB1 and TCON, whereas TPB2 showed only 38 families of bacteria. Dose-dependent enhancement of bacteria was noted in Rhodobacteraceae and Bacillaceae, whereas dose-dependent decreased abundance was recorded in Reyranellaceae, Bdellovibrionaceae, and Pseudomonadaceae. Furthermore, it was observed that Fusobacteraceae and Hypomicrobiaceae were increased in both the treated groups. The higher dose group (TPB2) showed a significantly higher abundance of Gemmataceae and Isosphaeraceae, whereas a lower abundance was recorded in Rhizobiaceae and Rubritaleaceae (Figure 2D).

#### 3.1.5 Genus

The genus-level composition analysis of the top 50 hits revealed that healthy control tilapia fish harbored 44 genera of bacteria in their gut system, with the highest dominance of Cetobacterium (4.94%), followed by *Legionella* (4.0%), Alpha cluster (3.41%), Pirellula (3.14%), Reyranella (2.23%), Rhodobacter (1.76%), *Bdellovibrio* (1.53%), *Pseudomonas* (1.41%), Luteolibacter (1.41%), and *Bacillus* (1.29%). The comparison showed that Hydrogenophaga and Mythylocystis were absent in the control fish but present in both treatment groups. In the TPB2, six genera, namely, *Chlamydia*, Neochlamydia, *Micrococcus*, Novosphyngobium, *Streptomyces*, and Sulfurospirillium, were absent. Therefore, a total of 38 genera could be detected in this group, whereas TPB1 showed 46 genera. A dose-dependent increase in the population of bacteria was noted in the genus *Bacillus*, Rhodobacter, and Fimbriglobus, whereas a reduction was recorded in Cetobacterium, Reyranella, *Pseudomonas*, and *Acinetobacter* treatment groups. The treatment with a higher dose of prebiotics further reduced the population of *Legionella*, Luteolibacter, and *Mycobacterium* but enhanced the *Staphylococcus* and Singulisphaera genus of gut microbes (Figure 2E).

To understand the microbiome diversity, we also calculated Shannon and Chao1 diversity indices. Results of one-way ANOVA and Tukey posthoc HSD analysis revealed statistically significant differences in the mean Shannon Diversity index (F>Fcrit; *p* < 0.001) and Chao1 index (F>Fcrit; *p* < 0.001) when compared among samples.

### 3.2 Presence absence analysis

To understand the differential abundance of OTUs among samples, we drew Venn diagrams for various taxonomic levels (Figures 2A–E). Closer scrutiny of the figures revealed an almost uniform OTU distribution for samples TPB1 and TCON. However, considerable differences were observed in the OTU distributions for sample TPB2.

### 3.3 Prediction of functionality

In terms of carbohydrate metabolism pathways, dose-dependent enhancement was recorded for glycolysis V, which is the super pathway of glycol metabolism and degradation, glucose and xylose degradation, glycolysis II, and sulfoglycolysis. Glycogen biosynthesis I and glycolysis III were downregulated compared to the control group (TCON). Some of the other pathways, such as glycogen degradation, glucose degradation, and glycogen degradation II, were found to be activated by a lower dose (TPB1) of prebiotics, followed by reduced activity with a higher dose (TPB2).

Upregulating trends in arginine, ornithine, and proline conversion and degradation of L-arginine and L-ornithine, putrescine, and the 4-aminobutanoate pathway in a dose-dependent manner were noticed. Heme biosynthesis from glycine, L-arginine biosynthesis I, II, III, IV, L-serine, glycine biosynthesis I, and glycine betaine degradation I were upregulated at a lower dose compared to the control group and a higher dose of prebiotic treatment.

Aromatic amino acid biosynthesis, L-arginine biosynthesis, II arginine, polyamine biosynthesis, branched amino acid biosynthesis, mixed acid fermentation, and tetrapyrrole biosynthesis II were decreased in a dose-dependent manner.

**FIGURE 3:**
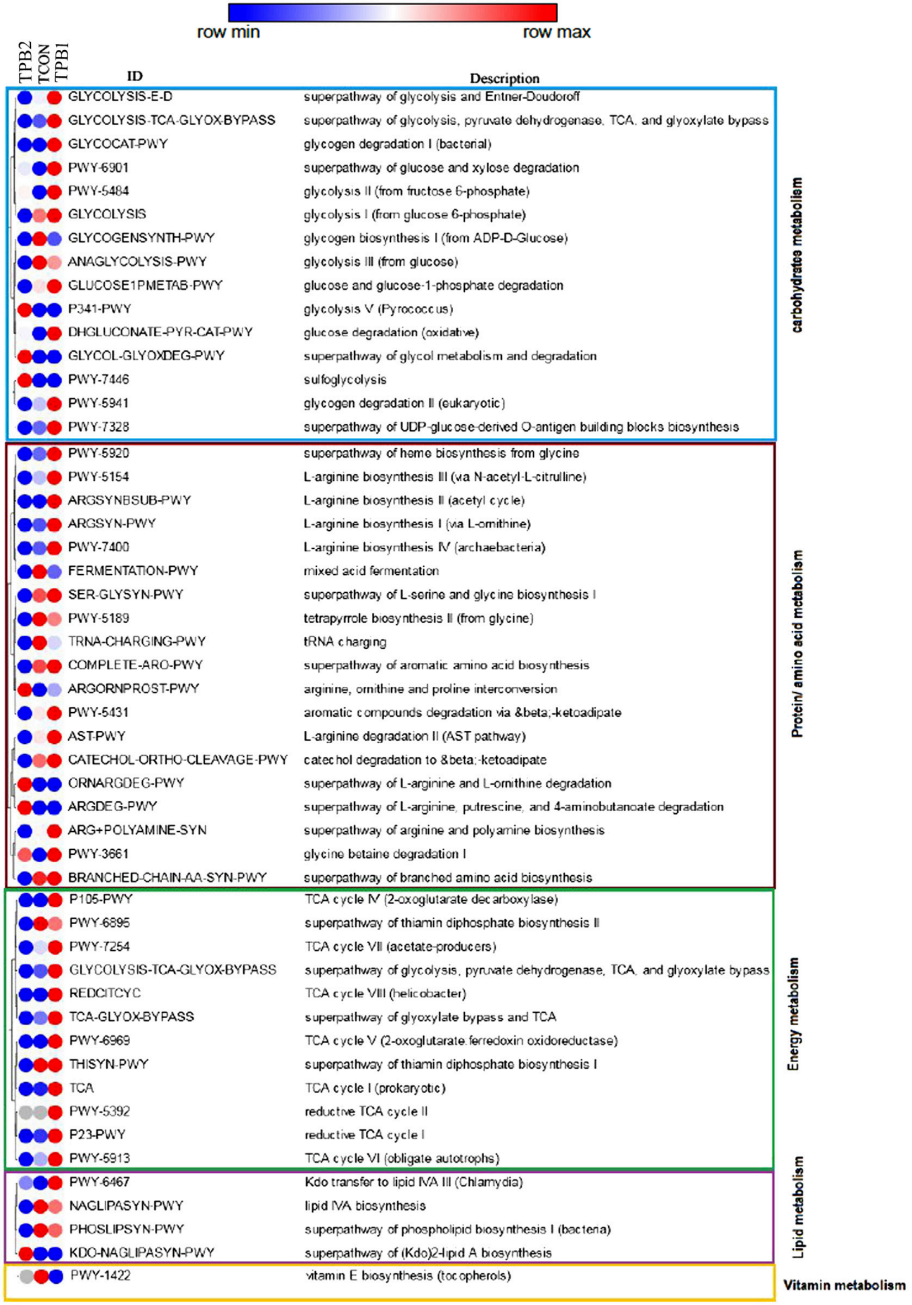
Comparison on prediction functionality of various metabolic pathways of gut microbiome in *O. niloticus* treated with prebiotics. [TCON–Control diet; TPB1—0.5% Aloe vera extract in the feed & TPB2—0.75% Aloe vera extract in the feed].

The thiamine diphosphate biosynthesis pathway was found to be downregulated in a dose-dependent manner, whereas the rest of the pathways, including Tricarboxylic acid cycle (TCA) cycle I, IV, VI, and VIII, and reductive TCA cycle I and II, were upregulated in the lower dose of prebiotics followed by downregulation at higher doses. A dose-dependent decreasing trend of pathway activities was recorded for lipid IVA biosynthesis and phospholipid biosynthesis I, whereas a reverse trend was seen for the super pathway of 2-lipid A biosynthesis. The lower dose group had a greater upregulation of the lipid IVA III pathway than the higher dose and control groups. The vitamin E biosynthesis pathway was found to be downregulated in both treatment groups when compared to the control group (Figure 3).

## 4 Discussion

The gut microbiome has been identified as a physiological and immune modulator, producing thousands of metabolites, and thus regulating the host’s health and sustenance. The diverse growth environment of terrestrial and aquatic animals causes a striking difference in the composition and structure of microbiota in well-studied mammals and other animals (Ni et al., 2014; Xiong et al., 2019). In contrast to terrestrial vertebrates, aerobic, facultative, anaerobic, and obligate anaerobic bacteria are the principal colonizers of the gastrointestinal tract of fishes (Opiyo et al., 2019). Bacterial cell count and concentration may also vary among human, rodent, and piscine populations. It is reported that fish gut harbors 10^7^–10^11^ bacteria g^−1^ of intestinal content (Nayak, 2010).

The impact of the presence and absence of some specific bacteria and their role in the innate immune system of a particular host is the functional dimension of the host-microbe interaction. The influence of gut microbiota in fish is reported to be similar to that in mammals concerning feeding behavior and physiology, secretion of enzymes, hormones, and metabolites (Yang and Chiu, 2017), such as butyrate, acetate, indole, and short-chain fatty acids (Duca et al., 2012; Zhang and Davies, 2016). Previous studies on fish gut microbiota and its diversity were reported in Lactobacillus-supplemented zebrafish, which showed a reduced appetite (Falcinelli et al., 2016), whereas Japanese flounders supplemented with *Bacillus clausii* showed enhanced growth rates with improved feed efficiency (Ye et al., 2011). The metabolism of carbohydrates, amino acids, and lipids is found to be influenced by the compositional changes of the gut microbiota of grass carp (Ni and Yu, 2013). Diet-induced alterations in gut microbiota composition also influence fatty acid absorption by the epithelium of zebrafish (Forsatkar et al., 2017).

The host’s stress adaptation and its associated sequelae are also mediated to a greater extent by the implication of its gut microbiota through energy homeostasis (Dehler et al., 2017). Fish with upset gut microbiota had disrupted energy homeostasis and enhanced stress hormones, as found in rainbow trout and goldfish (De Pedro et al., 1997; Ortega et al., 2013; Garcia-Reyero, 2018).

Previous studies (Ray et al., 2017; Zhu et al., 2020) have investigated how environmental factors and dietary composition affect the gut microbiota structure of Nile tilapia when fed with microbial community-fed diets supplemented with Previda^®^ and Saponin. Various water parameters, such as salinity and temperature, play an essential role in the gut microbiota composition of fish (Al-Harbi and Uddin, 2004; Ringø and Song, 2016). Intestinal dysbiosis biases, reduced immunity, and increased susceptibility to pathogenic organisms are associated with a wide range of pollutants such as heavy metals, pesticides, and antibiotics in the aquatic system (Li et al., 2016; Stephens et al., 2016; Butt and Volkoff, 2019).

Recently, a few studies have been carried out to investigate the gut microbiome structure of tilapia at different life stages (Giatsis et al., 2014; Koo et al., 2017). One study reported the microbiome profile of tilapia (Genetically Improved Farmed Tilapia, GIFT) in phylum categories predominantly belonging to Fusobacteria, Bacteroidetes, Proteobacteria, and Firmicutes. The role of gut microbiota in fish reproduction was recorded (Fan et al., 2017; Ray et al., 2017) where the phyla Proteobacteria, Firmicutes, and Actinobacteria with the highest count of *Fusobacterium* (≥81%) were found dominating during the peak breeding period.

In our current study, we discovered that Proteobacteria was the most dominant phylum, with Verrucomicrobia and *Chlamydia* as minor phyla. Our finding is consistent with that of Li et al. (2017), who reported Proteobacteria, Firmicutes, Fusobacteria, and Bacteroidetes as the dominant phyla in the gut of a large yellow croaker (*Pseudosciaenacrocea*).

The proper functioning of the host immune system is the result of healthy host-microbiome interaction, which otherwise when incurred by unfavorable environmental challenges, causes mild to severe diseases. Gut microbiota and the gut mucosal immune system work together to promote immunity in fish by maturing and developing gut-associated lymphoid tissue and secreting antimicrobial peptides (Kim et al., 2017; Wang et al., 2018).

Prebiotics and probiotics can play an indispensable role in maintaining optimal health and treating chronic diseases as a consequence of variations in the gut microbiota composition (Lin et al., 2014). During physical and biological stress, intensively cultured fish compromise their innate immunity and become easily vulnerable to diseases. This can be effectively addressed by using suitable feed supplements, such as prebiotics and probiotics, instead of any growth-promoting agents or antibiotics. Prebiotics are generally considered as feed ingredients capable of favoring the growth of beneficial microbes in the gut environment (probiotics) of the animal host. The supplemented diet can not only help to overcome diseases but also modulate the immune system for better growth of the host organism (Amenyogbe et al., 2020). These are mostly oligosaccharides of plant or microbial origin. The compounds enhance the probiotics’ population in the gut and also potentiate the immune system. Thus, supplementation with a suitable combination of prebiotics and probiotics is believed to be beneficial for fish health and growth by manipulating their gut microbiota. The storage polysaccharide, acetylated glucomannan, present mostly in the leaf of *A. vera* has numerous immunomodulatory, antimicrobial, anti-inflammatory and anti-cancer, properties (He et al., 2005; Jani et al., 2007). The acetylated β-(1→4)-D-mannosyl residues present in the mannans of *A. vera* counts significantly in the therapeutic constituents of the plant (Boudreau and Beland, 2006). Lesser amounts of arabinose, galactan and galactogalacturan units are the other biologically active polysaccharides present in the leaf gel of the plant (Ni et al., 2004).

The present investigation recorded that prebiotics at a dose of 0.5% of feed, showed two more numbers of the bacterial genus (Hydrogenophaga) and three more numbers of species (*Bacillus*, Circulans, Gemmata *spp*., and *Pedicoccuspentosaceus*) that were absent in the control group of tilapia fish. The higher dose of prebiotics resulted in a reduced number of bacteria at the phylum, class, family, genus, and species levels with an increase in the number of beneficial bacteria, which indicated that the selection of a suitable dose of prebiotics is necessary for the optimal manipulation of gut microflora to achieve beneficial effects. Conventional culture methods employed for the investigation of the GI microbiota of fishes were found to have limitations due to their dependency on culture conditions and the type of media used (Spanggaard et al., 2000). Besides, these methods consume more time with less accuracy in identifying bacterial isolates. Due to the low cultivability of the fish gut microbiome, less than 0.1% of the total microbial community could be detected in some fish (Zhou et al., 2014; de Souza et al., 2020). However, recent advances in molecular-based technologies and bioinformatics analysis have substantially redefined gut microbiome studies, with a wide array of data showing the elaborate elucidation and interaction of the structure, distribution, and diversity of bacterial phyla within the fish gut (Parma et al., 2016; Wang et al., 2018).

Microbial metabolic pathway analysis using PICRUSt is mostly suggested in association with carbohydrates, protein, amino acids, energy metabolism, membrane transport, nutrient digestion, immune function, and xenobiotic metabolism within the fish gut (García-Márquez et al., 2022). Feeding habits and trophic levels of fish are important factors to determine parts of the gut microbe community. Hence, variation in feeding strategies could be an important approach to achieving the beneficial effects of gut microbes on fish health. Prebiotics have been shown to modulate the microbial community, which improves feed digestion and metabolism of important nutrients and molecules (Pratoomyot et al., 2010). Our findings suggest that plant polysaccharide-based prebiotics can modulate the microbial population of the tilapia gut microbiome and subsequently regulate various metabolic pathways involved in physiology, homeostasis, health, immunity, and disease resistance. Treatment with prebiotics could also improve carbohydrate metabolism through gaining firmicutes and bacilli. These two groups of bacteria are responsible for the breakdown of complex polysaccharides and carbohydrates (Maji et al., 2022). The KEGG pathway analysis also indicated an increase in carbohydrate metabolism for glycolysis V sulfoglycolysis and glycol metabolism. Feeding insect meals to fish was previously reported to induce pentose and glucuronate interconversion (Panteli et al., 2021). Most of the amino acid and protein metabolism pathways were found to be upregulated at a lower dosage of the prebiotics, indicating an optimum balance of the microbial community at a certain dosage level. The same observation was also recorded for energy metabolism, including the TCA cycle. As recorded earlier, Cetobacterium and Bacteroidetes are generally linked with protein digestion and synthesis (Liu et al., 2016; Xu et al., 2019), whereas *Lactobacillus* and *Bacteroides* contribute to glucose and lipid metabolism. In the present study, a decrease in the abundance of Proteobacteria and Gamma Proteobacteria in the higher dose group could be the cause of downregulation in some protein and amino acid synthesis pathways.

## 5 Conclusion

Taken together, our current investigation revealed the structure of the gut microbiome of *O. niloticus* under Indian farming conditions, which are mostly tropical in nature. Moreover, the study highlighted the potential for modulation of the gut microbiome of tilapia through polysaccharide-based prebiotic feeding for efficient nutritional and gut health management and enhanced productivity. The modulated microbial abundance further reflected various metabolic pathways, hence explaining the effective biochemical mechanism.

## Data Availability

The data presented in the study are deposited in the NCBI database repository, accession number PRJNA977195. The data can be accessed via the link: http://www.ncbi.nlm.nih.gov/bioproject/977195.

## Abbreviations

OTU, Operational Taxonomic Unit; cfu, Colony forming unit; GIFT, Genetically Improved Farmed Tilapia; GALT, Gut associated lymphoid tissue; HSD, Honest significant difference; BHT, Butylated hydroxytoulene.
