# Supplementary material for: Microbiome analysis reveals potential for modulation of gut microbiota through polysaccharide-based prebiotic feeding in Oreochromis niloticus (Linnaeus, 1758)
Source: Front Physiol. 2023 Jun 8;14:1168284. doi: 10.3389/fphys.2023.1168284 (PMC10285058; doi:10.3389/fphys.2023.1168284)
Supplement: Supplementary file 1 [file DataSheet1.doc]

Supplementary Material

Microbiome analysis reveals potential for modulation of gut microbiota through polysaccharide-based prebiotic feeding in *Oreochromis niloticus* (Linnaeus, 1758)

**Asit K. Bera^1^, Hemanta Chowdhury^1^, Sandeep Ghatak^2^, Ramesh C. Malick^1^, Nabanita Chakraborty^1^, Hirak J. Chakraborty^1^, Himanshu S. Swain^1^, M. A. Hassan^1^ and Basanta K. Das^1^***

*** Correspondence:** Dr. Basanta Kumar Das, basantakumard@gmail.com

## Supplementary Figure


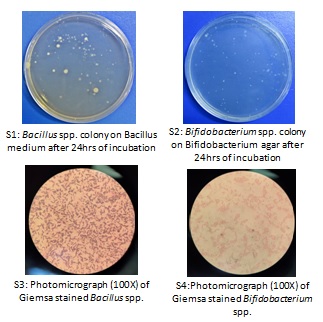


**Supplementary Figure 1.** *Bacillus* spp and *Bifidobacterium* spp. grown in culture medium (S1 & S2) and colonies in photomicrograph (100X) (S3 & S4).
